# Biosensor-Based Directed Evolution of Methanol Dehydrogenase from *Lysinibacillus xylanilyticus*

**DOI:** 10.3390/ijms22031471

**Published:** 2021-02-02

**Authors:** Thien-Kim Le, Su-Bin Ju, Hye-Won Lee, Jin-Young Lee, So-Hyung Oh, Kil-Koang Kwon, Bong-Hyun Sung, Seung-Goo Lee, Soo-Jin Yeom

**Affiliations:** 1 School of Biological Sciences and Biotechnology, Graduate School, Chonnam National University, Yongbong-ro 77, Gwangju 61186, Korea; thienkim.1611@gmail.com (T.-K.L.); jsb9706@naver.com (S.-B.J.); 2 School of Biological Sciences and Technology, Chonnam National University, Gwangju 61186, Korea; 3 Synthetic Biology and Bioengineering Research Center, Korea Research Institute of Bioscience and Biotechnology, Daejeon 34141, Korea; hlee@kribb.re.kr (H.-W.L.); jylee84@kribb.re.kr (J.-Y.L.); sohyungoh@kribb.re.kr (S.-H.O.); kkkwon@kribb.re.kr (K.-K.K.); bhsung@kribb.re.kr (B.-H.S.); 4 Department of Biosystems and Bioengineering, KRIBB School of Biotechnology, University of Science & Technology, Daejeon 34113, Korea

**Keywords:** synthetic methylotrophy, methanol dehydrogenase, *Lysinibacillus xylanilyticus*, biosensor, screening

## Abstract

Methanol dehydrogenase (Mdh), is a crucial enzyme for utilizing methane and methanol as carbon and energy sources in methylotrophy and synthetic methylotrophy. Engineering of Mdh, especially NAD-dependent Mdh, has thus been actively investigated to enhance methanol conversion. However, its poor catalytic activity and low methanol affinity limit its wider application. In this study, we applied a transcriptional factor-based biosensor for the direct evolution of Mdh from *Lysinibacillus xylanilyticus* (Lxmdh), which has a relatively high turnover rate and low *K*_M_ value compared to other wild-type NAD-dependent Mdhs. A random mutant library of *Lxmdh* was constructed in *Escherichia coli* and was screened using formaldehyde-detectable biosensors by incubation with low methanol concentrations. Positive clones showing higher fluorescence were selected by fluorescence-activated cell sorting (FACS) system, and their catalytic activities toward methanol were evaluated. The successfully isolated mutants E396V, K318N, and K46E showed high activity, particularly at very low methanol concentrations. In kinetic analysis, mutant E396V, K318N, and K46E had superior methanol conversion efficiency, with 79-, 23-, and 3-fold improvements compared to the wild-type, respectively. These mutant enzymes could thus be useful for engineering synthetic methylotrophy and for enhancing methanol conversion to various useful products.

## 1. Introduction

Methanol dehydrogenase (Mdh) catalyzes methanol oxidation to formaldehyde with two electrons and 2H^+^. Methylotrophs are a diverse group of microorganisms that can use reduced C1 compounds such as methanol or methane as carbon sources for their growth [1]. Depending on the electron acceptors, there are three types of Mdh: PQQ (pyrroloquinoline quinone)-dependent MDH, NAD-dependent MDH, and O_2_-dependent AOD (alcohol oxidase). In particular, NAD-dependent MDH may be the best candidate for recombinant-based synthetic methylotrophy because it is a single subunit of an enzyme, and it can function under both aerobic and anaerobic conditions to generate reducing equivalents (NADH), which in turn can help promote strain growth [2]. 

With increasing attention to synthetic methylotrophy as a practical methanol-based biomanufacturing, interest in methanol dehydrogenase has also increased; however, poor catalytic activity and low methanol affinity of Mdh is a major limitation in the synthetic methylotrophy development and its applications. Although methanol utilization studies have been previously progressed toward establishing *Escherichia coli* (*E. coli*) as a synthetic methylotrophy by applying NAD-dependent Mdh from *Bacillus stearothermophilus*, *Bacillus methanolicus*, and *Cupriavidus necator* [3,4,5], recombinant *E. coli* cannot utilize methanol as a sole carbon source because of low methanol affinity of Mdh. The catalytic activity of Mdh from *B. methanolicus* has been reported to be dramatically enhanced using an endogenous activator protein (ACT); however, the detailed mechanism for Mdh activation remains unclear [6,7].

To enable metabolic engineering for assimilating methanol as a carbon source in synthetic methylotrophy, a new Mdh without ACT and with high activity under mesophilic or thermophilic conditions is required. In a previous study, we developed a new ACT-independent Mdh from *Lysinibacillus xylanilyticus* (Lxmdh), which has high activity. Additionally, rational approach-based mutation studies resulted in mutants with higher turnover rate compared to the wild-type, even though the *K*_M_ values were comparatively increased [8]. However, the improvement of Lxmdh catalytic activity and substrate affinity toward low-concentration methanol remains a challenge in the development of Mdh-driven synthetic methylotrophs.

Transcriptional factor (TF)-based biosensors are powerful tools that can be used to increase enzyme activity or detect new strains and enzymes. In this system, fluorescence signals are released from specific TF responsible cells fluorescence, in the presence of specific metabolites or in vivo enzyme activity. Various TF-based biosensors have been developed for the discovery of novel biocatalysts and enzyme engineering using a fluorescence-activated cell sorting (FACS)-based high-throughput screening system [9,10,11,12,13,14,15,16,17,18]. In particular, some TF-based biosensors to detect methanol (MxaYZ-TCS) have been developed for screening C1-converting enzymes and for developing biochemical-producing recombinant microorganisms [18]. Previously, we developed formaldehyde-detectable genetic enzyme screening system (Frm-GESS) that can be applied for the direct evolution of Mdh based on FACS screening systems [19]. This TF-based screening system was highly appropriate as a screening technique to engineer C1-converting enzymes such as Mdh.

In this study, we applied a random mutagenesis-based directed evolution method to further engineer Lxmdh to achieve high catalytic activity and affinity for methanol. For directed evolution, we investigated a Frm-GESS as a high-throughput screening (HTS) platform that enables the detection of Lxmdh activity by releasing formaldehyde-dependent fluorescence. The results indicated that this method is highly appropriate as a screening technique to explore libraries for catalysis of methanol oxidation in living cells. The study also presents mutants that may be potentially helpful for the development of synthetic methylotrophy. 

## 2. Results and Discussion

### 2.1. Biosensor-Based Screening of the Lxmdh Library

**Figure 1:**
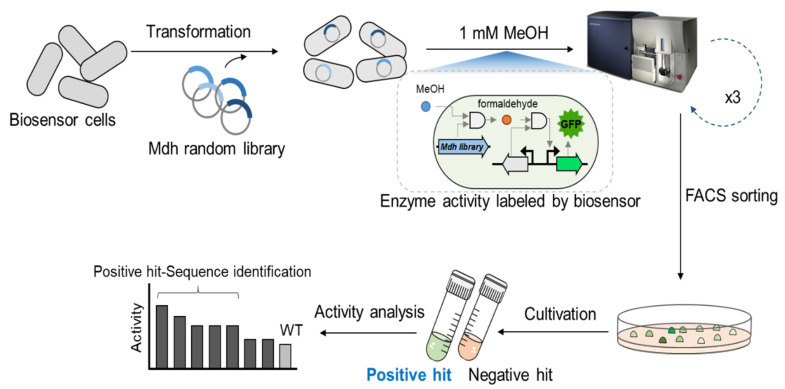
Schematic of the screening procedure for methanol dehydrogenase (Mdh) evolution using a formaldehyde-detectable transcription factor-based biosensor and random library.

**Figure 2:**
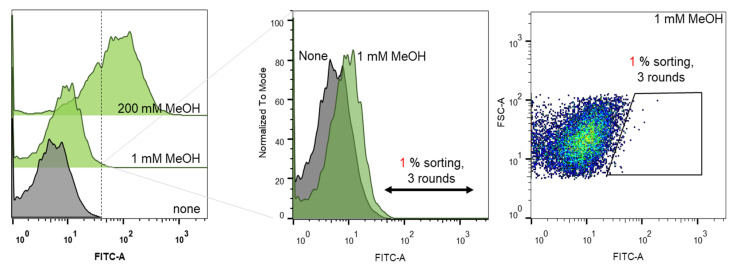
Flow cytometric analysis of cells harboring the Lxmdh mutant library at different concentrations of methanol, i.e., 0, 1 and 200 mM.

To engineer Lxmdh for high catalytic activity and methanol affinity, we developed a TF biosensor-based screening strategy, as shown in Figure 1. The randomly mutagenized library of Lxmdh was constructed and applied to the formaldehyde-detectable genetic enzyme screening system (Frm-GESS) with 1 mM methanol as the substrate. Upon the formation of formaldehyde according to Mdh activity, the biosensor cells expressed fluorescent proteins, and were subsequently analyzed and sorted using flow cytometry. Three rounds of screening were performed to isolate the putative Lxmdh mutants, whose fluorescent intensities were the highest 1% of total cells analyzed during screening (Figure 2). To minimize false positive rates, the sorting threshold was defined in the consideration of the size distribution of microbial cells.

Next, after cultivation in solid and liquid media, we re-analyzed the selected putative mutants as populations, and verified that the population average of fluorescence signals increased (Figure 1 and Figure S1). As shown in Supplementary Materials Figure S1, among 81 Frm-GESS with Lxmdh variants, 28 indeed showed high fluorescent responses. DNA sequence analysis of the Lxmdh variant revealed that the following four mutants were enriched and derived from the isolates: C364G, K46E+E314G, K318N+E396V, and V388I (Figure S1). 

**Figure 3:**
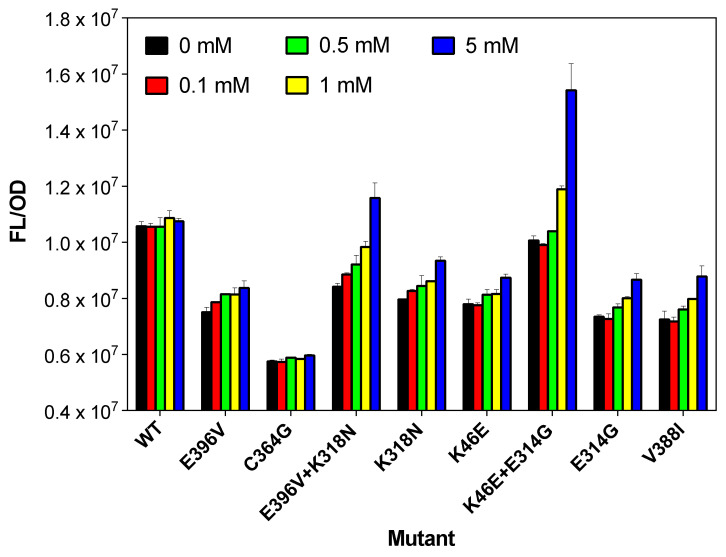
Fluorescence response of cells with pFrm-GESS and Lxmdh mutants to methanol. Cells harboring the formaldehyde biosensor plasmid pFrm-GESS and the Lxmdh variants were incubated with different concentrations of methanol in LB for 8 h.

Subsequently, site-directed mutagenesis of K46E, E314G, K318N, and E396V from double mutants was performed to determine the role of each residue except single mutants such as C364G and V388I. Finally, eight mutants (E396V, C364G, E396V+K318N, K318N, K46E, K46E+E314G, E314G, and V388I (mutation points shown in Figure S2) showed higher fluorescence/optical density (FL/OD) values compared with the wild-type when tested with various concentrations of methanol (0–5 mM) (Figure 3).

Our Frm-GESS can detect released formaldehyde from methanol based on Mdh activity. Thus, in the case of wild-type cells, fluorescence response is limited at over 1 mM of methanol due to a high km value. However, mutant cells show high increased fluorescence response at 0.1–0.5 mM methanol, which means that mutant enzymes have high affinity and activity toward low concentrations of methanol (Figure 3). With increasing methanol concentration, the FL/OD values of the wild-type strain showed no difference. In contrast, except for the C364G mutant, all seven mutants showed dose-dependent response of methanol. In particular, the E396V+K318N, K318N, K46E+E314G, E314G, and V388I mutants showed higher fluorescence levels in the presence of methanol. The highest changing in the FL/OD value was 1.52-fold (from 1.01 × 10^7^ to 1.54 × 10^7^) for the K46E+E314G mutant. The E396V+K318N mutant also exhibited an impressive change in the FL/OD value (1.38-fold, from 0.84 × 10^7^ in the absence of methanol to 1.16 × 10^7^ with 5 mM of methanol). For K318N, K46E, E314G, and V388I mutants, the variation in FL/OD values was similar, 1.12–1.21-fold (from 0.72–0.80 × 10^7^ to 0.87–0.93 × 10^7^). These results demonstrate the successful isolation of putative Lxmdh mutants using high-throughput Frm-GESS. 

### 2.2. Comparison of Enzyme Activity between the Wild-Type and Its Mutants

Nine Lxmdh enzymes (including the wild-type and eight mutants, V388I, C364G, E396V, E396V+K318N, K318N, E314G, K46E+E314G, K46E) were purified using a His-tag column. A pre-stained marker protein, crude extract, and purified enzymes were loaded on an SDS-PAGE gel for molecular weight analysis (Figure S3).

**Figure 4:**
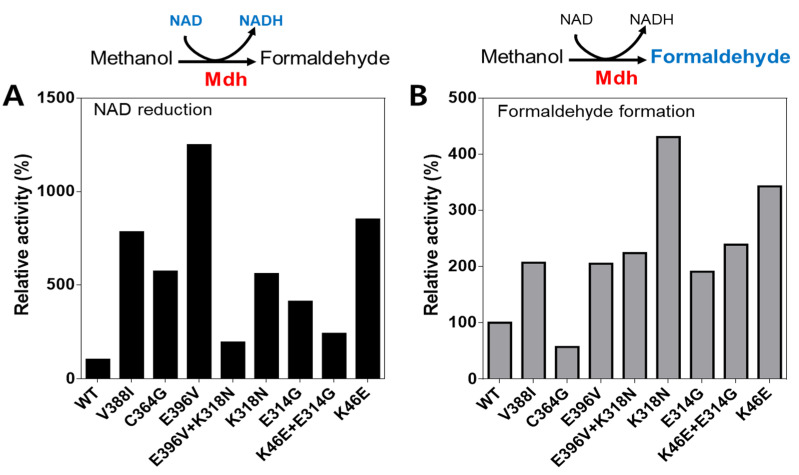
Comparison of enzyme activity between the wild-type (WT) and its mutants by the NAD reduction rate (**A**) and the concentration of formaldehyde generated from methanol by enzymes (**B**). The reaction mixture included 0.05 mg/mL protein, 10 mM NAD^+^, 5 mM Mg^2+^, and 100 mM methanol in 50 mM CHES (pH 9). The NAD reduction rates were monitored at RT (room temperature) for a short time (60 s) by spectrometer. For detection of the released formaldehyde concentration, samples were incubated at 55 °C for 10 min, and subjected to HPLC as described in the text.

The NAD reduction rates of the wild-type and its mutants were then compared to find the best candidates for the MeOH:NAD reaction (Figure 4A). Among the eight mutant enzymes tested, only the K46E+E314G mutant enzyme did not show any activity, whereas the seven remaining mutant enzymes showed a higher NAD reduction rate than the wild-type enzyme. Moreover, the mutants V388I, E396V, and K46E exhibited the highest NAD reduction rates (19.9, 31.7, and 21.6 (m)U/mg, respectively). The NAD reduction rate of E396V was 13-fold higher than that of the wild-type (2.5 (m)U/mg). Furthermore, the rates of C364G, K318N, and E314G mutants were similar (13.1, 14.5, 14.2, and 10.4, respectively). Importantly, among the eight mutant enzymes, the lowest value, of 4.9 (m)U/mg, was demonstrated by the E396V+K318N mutant.

To determine whether the Lxmdh mutant enzyme could use methanol as a substrate, the rate at which the wild-type and its eight mutants formed formaldehyde was analyzed by HPLC (Figure 4B). Seven of the mutant enzymes were found to demonstrate higher activity than the wild-type. The highest concentration of formaldehyde was formed by the K318N mutant, with a 4.3-fold increase compared to the wild-type. Interestingly, the K46E mutant also showed high catalytic activity that was comparable to that of the wild-type (3.4-fold). The V388I, E396V, E396V+K318N, E314G, and K46E+E314G mutants had similar rates, showing around two-fold increases compared to the wild-type.

Consequently, three mutants (E396V, K318N, and K46E) were selected for additional experiments, and their enzyme activity was compared depending on the methanol concentration and kinetic analyses.

### 2.3. Enzyme Activity by Concentration of Generated Formaldehyde with Respect to Methanol Concentration

**Figure 5:**
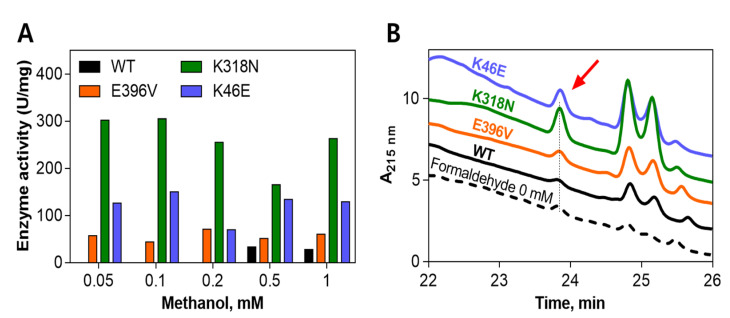
Comparison of enzyme activity among the wild-type and its mutants (E396V, K318N and K46E) by the concentration of generated formaldehyde with respect to methanol concentration (**A**), and the HPLC trace of each sample with 0.05 mM of MeOH as the substrate (**B**). The samples in 40 µL CHES buffer (50 mM, pH 9), containing 0.05 mg/mL protein, 10 mM NAD^+^, 5 mM of Mg^2+^ and methanol (from 0.05 to 1 mM), were incubated at 55 °C for 10 min. The formaldehyde formation rate was determined by HPLC as described in the text.

As mentioned above, the mutants E396V, K318N, and K46E, which exhibited high activity (in both the NAD reduction rate and product formation rate), were chosen for reaction with a range of methanol concentrations compared to the wild-type (Figure 5). All mutants tested here showed formaldehyde generation as a product by HPLC analysis, even at a low methanol concentration (0.05 mM). The concentration of formaldehyde formed by the K318N mutant was the highest, at 305 U/mg with 0.1 mM of methanol as the substrate. The mutants E396V and K46E showed lower values compared to the K318N mutant, with their highest values being 71 and 150 U/mg, respectively. On the contrary, the wild-type showed activity only at 0.5 and 1 mM of methanol with low values (33 and 28 U/mg, respectively).

### 2.4. Kinetic Parameters of the Wild-Type and Mutant Enzymes

Table 1 shows the steady-state kinetics of formaldehyde generation from methanol by Lxmdh wild-type and its mutants (E396V, E396V+K318N, K318N, and K46E). The wild-type showed the highest *k*_cat_ value (2.0 min^−1^). Compared to the value of the wild-type, the *k*_cat_ value of its mutants was low. Mutants K318N and K46E had similar *k*_cat_ values of 1.6 and 1.7 min^−1^, respectively. In addition, the lowest *k*_cat_ value detected belonged to mutant E396V (1.2 min^−1^). The double mutant, E396V+K318N, had a higher value (1.3 min^−1^) compared to the single mutant E396V. However, this was still lower than that of the single mutant K318N.

Most importantly, the mutant E396V showed the lowest *K*_M_ value (0.010 mM, Table 1), which was much lower than that of wild-type (1.311 mM): by 131-fold. Therefore, the catalytic efficiency (*k*_cat_/*K*_M_) of formaldehyde formation by the mutant E396V was 120 min^−1^.mM^−1^, which was more efficient than that of the wild-type, by 79-fold. Another mutant, K318N, showed a *K*_M_ value of 0.046 mM, which was lower than that of the wild-type by 29-fold. The double mutant E396V+K318N and the mutant K46E also showed low *K*_M_ values (0.233 and 0.372 mM, respectively).

In summary, although the *k*_cat_ value of the wild-type was the highest, all the mutants showed a lower *K*_M_ value than that of the wild-type. Thus, the catalytic efficiency of formaldehyde formation by the Lxmdh mutants was higher than that of the wild-type by 3–79-fold. 

The wild-type Lxmdh has been reported to demonstrate relatively high substrate specificity toward methanol when compared with Mdh enzymes from *C. necator*, *B. methanolicus*, and *B. stearothermophilus* [4,7,8]. The reported *K*_M_ value of Lxmdh for methanol was 3.23 mM [8]. This value was quite low compared to that of Mdh and Mdh2 derived from *B. methanolicus* (9 mM) [20], Mdh from *C. necator* (132 mM) [4] and Mdh, Mdh2, and Mdh3 from *B. methanolicus* (170–360 mM) [7]. Two types (I and II) of Mdh from *Methylophaga aminisulfidivorans* have been reported to have *K*_M_ values of 50.3 µM and 13.0 µM using the cytochrome *c*_L_ reduction assay system [21], but there is no report for type III Mdh.

Furthermore, the *K*_M_ of E396V at 0.01 mM was much lower than that of the wild-type (by 131-fold). Our findings can thus provide promising Mdh enzymes with the lowest *K*_M_ value and highest catalytic efficiency among other Mdhs. These mutants can be applied to synthetic methylotrophy, which is an important research area because valuable chemicals can be produced through C1 assimilation.

## 3. Materials and Methods

### 3.1. Chemicals and Materials

LB media, ampicillin and kanamycin were purchased from MBcell (SeoCho-Gu, Korea). Methanol, formaldehyde, NADPH, and other chemicals were purchased from Sigma-Aldrich (St. Louis, MO, USA). Restriction endonucleases and Phusion® High-Fidelity DNA Polymerase were purchased from New England Biolabs (Ipswich, MA, USA). Diversify PCR random mutagenesis kit and PrimeSTAR® Max DNA Polymerase, which were used for construction of Lxmdh mutant library, were purchased form Takara (Tokyo, Japan). The kits for PCR product purification and gel extraction were purchased from Promega (Madison, WI, USA). QIA prep Spin mini-prep kit for plasmid preparation and Ni-NTA Superflow columns 12 × 1.5 mL for protein purification were purchased form QIAGEN (Hilden, Germany). Oligonucleotides and gene synthesis services were provided by Macrogen (Seoul, Korea). All materials for SDS-PAGE were purchased from Bio-Rad (Hercules, CA, USA). Additionally, all DNA techniques were carried out following the standard protocols for molecular biology.

### 3.2. Strains and Plasmids

*E. coli* DH5α was used as a cloning host and for the expression of Lxmdh. The recombinant vector for constitutive Lxmdh expression was constructed using pET28a/Lxmdh as a template, which was cloned in a previous study [8]. The T7 promoter of pET28a/Lxmdh was replaced with the trc promoter to apply *E. coli* DH5α. The primers F-ctcgtataatgtgtgggggaattgtgagcggataac and R-ccggatgattaattgtcaaatttcgcgggatcgagatctcg were used for cloning pET/tac-Lxmdh. The DNA fragment of pET/tac-Lxmdh was amplified by PCR using Phusion® High-Fidelity DNA Polymerase (New England Biolabs, Ipswich, MA, USA), and was then assembled by the Blunting Kination Ligation method to construct pET/tac-Lxmdh. pFrm-GESS, a formaldehyde biosensor plasmid, was obtained from a previous study [19].

### 3.3. Construction of Lxmdh Mutant Library

To construct the library, we used pET/tac-Lxmdh which changed the T7 promoter to the tac promoter as a template. Then, the PCR fragment of Lxmdh was amplified by random mutagenesis PCR using primers (insert: F-ggtgccgcgcggcagccatatgtcagacgttctaaagc and R-gtaccatgggatccctcgagttaagaaagtgcgacagctt). Diversify PCR random mutagenesis kits (Takara, Kyoto, Japan) were used for constructing the Lxmdh mutant library, according to the manufacturer’s instructions. The buffer conditions of the random mutagenesis kits were selected for 2.7–4.6 mutations per 1000 bp of DNA (320–480 μM MnSO_4_, 40 μM dGTP). The pET vector part was also amplified using primers (vector: F-aagctgtcgcactttcttaactcgagggatcccatggtac and R-ttagaacgtctgacatatggctgccgcgcggca) using phusion tag polymerase (NEB). Finally, the amplified mutant library DNA of Lxmdh was ligated with the pET vector part using Gibson assembly, and then transformed by electroporation into pFrm-GESS biosensor cells, which were freshly cultured in LB/Amp medium. After electroporation, the mutant library (approximately 10^5^–10^6^ cells) was plated in an LB/Amp/Kan square plate, and after 16 h of incubation at 37 °C, the cultured cells were harvested using a scraper. The obtained cells were resuspended in LB/Amp/Kan medium containing 20% glycerol, adjusted to OD_600_ 4.0 by dilution of the harvested cells, aliquoted at 1 mL/cryotube vial, and stored at −80°C for further studies. 

### 3.4. Library Screening Using Flow Cytometry

The stored mutant library of Lxmdh in the pFrm-GESS biosensor cells was thawed at RT for 20 min; then, 300 μL of the cells were inoculated in 2.7 mL of LB/Amp/Kan medium. As a negative control, the pFrm-GESS biosensor cell containing wild-type Lxmdh was prepared using the same method. Cells were incubated at 37 °C, 200 rpm for 1 h; methanol (1 mM) was then added as a substrate, and further incubated for 1–3 h. For screening positive clones, 20 μL of cultured cells were diluted in 1 mL of PBS solution, and sorted using an FACS Aria II Flow Cytometer (BD Bioscience, Franklin Lakes, NJ, USA). Samples were sorted using a 70 μL nozzle, which is specifically suited for bacterial sorting. The population was gated by forward scatter and side scatter, and the GFP signal was compared with that of the control group; the data were analyzed using FlowJo software (Tree Star, Ashland, OR, USA). The positive clones in the mutant library, which had a relatively high fluorescence signal (upper 1%) of sfGFP, were collected into tubes containing 2 mL of LB medium, and then cultured at 37 °C and 200 rpm for 16 h. The FACS screening process was repeated twice for the enrichment of positive clones. After sorting, the positive cells were plated on the LB/Amp/Kan plate medium.

### 3.5. Screening the Lxmdh Library by Liquid-Phase Fluorescence Analyses

The stored mutant library of Lxmdh in the Frm-GESS biosensor was transformed by electroporation into *E. coli* DH5α cells. Colonies were picked to grow overnight in LB media containing 100 μg/mL ampicillin and 25 g/mL kanamycin at 37 °C, 200 rpm. For the first round of screening, the reaction was initiated by adding 1 mM of methanol to the medium in a 96-well plate.

For mutant fluorescence analysis in the liquid phase, single colonies of *E. coli* DH5α harboring the sorted mutant library were inoculated in 2 mL of LB medium containing 100 μg/mL ampicillin and 25 g/mL kanamycin. After cultivation at 37 °C for 8 h, 1% (*v*/*v*) of the seed culture was inoculated into 2 mL of fresh LB containing ampicillin and kanamycin along with 1–5 mM of methanol. The fluorescence intensity was measured after 16 h at 37 °C on a multi-label microplate reader (PerkinElmer, Waltham, MA, USA) at excitation and emission wavelengths of 485 and 535 nm, respectively.

### 3.6. Purification of Lxmdh

*E. coli* C2566 cells harboring pET28a-d-amino acid amidases were grown in LB media at 37 °C until they reached an OD_600_ of 0.4–0.5. The cells were then induced with 0.1 mM IPTG. The culture was transferred to 20 °C, incubated for 18 h, harvested by centrifugation at 3800 rpm and 4 °C for 20 min, and resuspended in lysis buffer (500 mM NaCl, 20 mM Tris pH 8.0). The cell extracts were then prepared by sonication, purified using Ni-NTA Superflow columns 12 × 1.5 mL (QIAGEN, Hilden, Germany), and then dialyzed with 50 mM CHES buffer (pH 9.5). The protein concentration was quantified using the Bradford method. The purified proteins were confirmed by SDS-PAGE.

### 3.7. Comparison of Enzyme Activity between the Wild-Type and Its Mutants

Nine mutants were selected from a biosensor-based screening of the Lxmdh library by the fold change in specific fluorescence (FL/OD) for comparing enzyme activity with the wild-type. The reaction mixture included 50 mM CHES buffer (pH 9.5), 10 mM NAD^+^, 5 mM Mg^2+^, and 0.05 mg/mL protein with 100 mM methanol as the substrate. The NAD^+^ reduction rate was measured using a UV–VIS Spectrometer (UV-1900, Shimazu, Kyoto, Japan) at room temperature. In this study, we analyzed formaldehyde formation from methanol by Lxmdh.

The reaction was stopped by adding 100 µL of 0.13 mM *O*-Benzylhydroxylamine (BnONH_2_) after 10 min. The mixture was incubated at room temperature for 10 min. After centrifugation and filtration, product formation was analyzed by HPLC and quantified by comparing their concentrations with those of standard compounds [22]. The results were analyzed using GraphPad Prism (GraphPad Software, San Diego, CA, USA). 

### 3.8. Enzyme Activity by the Concentration of Formaldehyde Generated with Respect to Methanol Concentration

The enzyme activity of wild-type and three chosen mutants (K318N, E396V, K46E) was determined in 40 µL of CHES buffer (50 mM, pH 9). The reaction mixtures contained 0.05 mg/mL protein, 10 mM NAD^+^, 5 mM of Mg^2+^, and methanol (from 0.05 to 1 mM). The samples were incubated at 55 °C for 10 min. The formaldehyde formation rate was determined by HPLC, as described above.

### 3.9. Determination of Kinetic Parameters of Wild-Type and Mutant Enzymes

To determine the steady-state kinetics (*k*_cat_ and *K*_M_) of each Lxmdh mutant, the reaction mixture contained 0.05 mg/mL of the mutant enzyme, 5 mM Mg^2+^, 3 mM NAD^+^, and a range of methanol concentrations (from 0 to 5 mM) in 40 µL of CHES buffer (50 mM, pH 9.5). The reaction mixtures were incubated at 55 °C for 10 min. The concentration of generated formaldehyde was detected by HPLC, as described above. The kinetic parameters (*k*_cat_ and *K*_M_) were calculated by Michaelis–Menten nonlinear regression analysis using GraphPad Prism.

### 3.10. Analytical Methods

For quantitative determination, formaldehyde in the reaction mixture was determined by HPLC (UV 215 nm) using an instrument fitted with a Gemini C18 column (4.6 × 250 mm, 5 µm; Phenomenex, Torrance, CA, USA), using 0.1% trifluoroacetic acid in DW (A) and 0.095% trifluoroacetic acid in ACN:DW (80:20, *v*/*v*) (B) as the mobile phase with a gradient from 10% to 100% for 30 min at 30 °C after derivatization with BnONH_2_, as described above. The reaction mixture (50 µL) for derivatization contained BnONH2 and was injected into the column [22].

## 4. Conclusions

C1 assimilation can produce high-value chemicals; therefore, development of synthetic methylotrophy harboring various C1 chemical converting enzymes will be an important research field. A TF-based biosensor could provide wide-ranging applications in the rapid screening of new C1 chemical converting enzymes. The first enzyme participating in methanol oxidation was Mdh as a key enzyme in synthetic methylotrophy. Lxmdh was reported in a previous study with higher activity and lower *K*_M_ toward methanol compared to that of other type III Mdhs. Nevertheless, the activity of Lxmdh was not sufficient for application to synthetic methylotrophy. To improve Lxmdh enzyme activity and substrate affinity for methanol, we contributed to the Lxmdh random library to formaldehyde-detectible biosensor-based screening systems. Various mutants were characterized by lower *K*_M_ and higher catalytic efficiency than the wild-type Lxmdh. In particular, we found mutant E396V with the highest catalytic efficiency of 79-fold greater compared with the wild-type. This mutant may thus potentially be helpful for the development of synthetic methylotrophy in the future.

## Figures and Tables

**Table 1:** Kinetic parameter of Lxmdh wild-type and its mutants with methanol as a substrate.

Enzyme	*k*_cat_ (min^−1^)	*K*_M_ (mM)	*k*_cat_/*K*_M_ (min^−1^.mM^−1^) (Relative Catalytic Efficiency)
Wild-type	2.0 ± 0.2	1.311 ± 0.406	1.526 (1)
E396V	1.2 ± 0.1	0.010 ± 0.003	120 (79)
E396V+K318N	1.3 ± 0.1	0.233 ± 0.107	5.579 (4)
K318N	1.6 ± 0.3	0.046 ± 0.072	34.78 (23)
K46E	1.7 ± 0.2	0.372 ± 0.231	4.570 (3)

## Data Availability

The raw data supporting the conclusions of this article will be made available by the authors, without undue reservation, to any qualified researcher.

## Supplementary Materials

The following are available online at https://www.mdpi.com/1422-0067/22/3/1471/s1. Figure S1. Fluorescence response of Frm-GESS with the Lxmdh variant in 200 mM methanol; Figure S2. Homology modelling of Lxmdh. The active site and mutation points of Lxmdh are represented as surface and thick stick, respectively; Figure S3. SDS-page analysis of Lxmdh wild-type and its mutants (V388I, H3-7F, C364G, E396V, E396V+K318N, K318N, E314G, K46E+E314G, and K46E). Pre-stained marker protein (75, 63, 48, 35, and 25 kDa), crude extract, and purified enzyme were loaded.

## References

[1] Quayle J.R., Ferenci T. (1978). Evolutionary aspects of autotrophy. Microbiol. Rev..

[2] Zhang W., Zhang T., Wu S., Wu M., Xin F., Dong W., Ma J., Zhang M., Jiang M. (2017). Guidance for engineering of synthetic methylotrophy based on methanol metabolism in methylotrophy. RSC Adv..

[3] Müller J.E., Meyer F., Litsanov B., Kiefer P., Potthoff E., Heux S., Quax W.J., Wendisch V.F., Brautaset T., Portais J.-C. (2015). Engineering Escherichia coli for methanol conversion. Metab. Eng..

[4] Wu T.-Y., Chen C.-T., Liu J.T.-J., Bogorad I.W., Damoiseaux R., Liao J.C. (2016). Characterization and evolution of an activator-independent methanol dehydrogenase from Cupriavidus necator N-1. Appl. Microbiol. Biotechnol..

[5] Whitaker W.B., Jones J.A., Bennett R.K., Gonzalez J.E., Vernacchio V.R., Collins S.M., Palmer M.A., Schmidt S., Antoniewicz M.R., Koffas M.A.G. (2017). Engineering the biological conversion of methanol to specialty chemicals in Escherichia coli. Metab. Eng..

[6] Hektor H.J., Kloosterman H., Dijkhuizen L. (2002). Identification of a Magnesium-dependent NAD(P)(H)-binding Domain in the Nicotinoprotein Methanol Dehydrogenase from Bacillus methanolicus. J. Biol. Chem..

[7] Krog A., Heggeset T.M.B., Müller J.E.N., Kupper C.E., Schneider O., Vorholt J.A., Ellingsen T.E., Brautaset T. (2013). Methylotrophic Bacillus methanolicus Encodes Two Chromosomal and One Plasmid Born NAD+ Dependent Methanol Dehydrogenase Paralogs with Different Catalytic and Biochemical Properties. PLoS ONE.

[8] Lee J.-Y., Park S.-H., Oh S.-H., Kwon K.K., Kim S.-J., Choi M., Rha E., Lee H., Lee D.-H., Sung B.H. (2020). Discovery and Biochemical Characterization of a Methanol Dehydrogenase from Lysinibacillus xylanilyticus. Front. Bioeng. Biotechnol..

[9] Kwon K.K., Lee D.-H., Kim S.J., Choi S.-L., Rha E., Yeom S.-J., Subhadra B., Lee J., Jeong K.J., Lee S.-G. (2018). Evolution of enzymes with new specificity by high-throughput screening using DmpR-based genetic circuits and multiple flow cytometry rounds. Sci. Rep..

[10] Choi S.-L., Rha E., Lee S.J., Kim H., Kwon K., Jeong Y.-S., Rhee Y.H., Song J.J., Kim H.-S., Lee S.-G. (2014). Toward a Generalized and High-throughput Enzyme Screening System Based on Artificial Genetic Circuits. ACS Synth. Biol..

[11] Kim H., Rha E., Seong W., Yeom S.-J., Lee D.-H., Lee S.-G. (2016). A Cell–Cell Communication-Based Screening System for Novel Microbes with Target Enzyme Activities. ACS Synth. Biol..

[12] Woolston B.M., Roth T., Kohale I., Liu D.R., Stephanopoulos G. (2018). Development of a formaldehyde biosensor with application to synthetic methylotrophy. Biotechnol. Bioeng..

[13] Yeom S.-J., Kim M., Kwon K.K., Fu Y., Rha E., Park S.-H., Lee H., Kim H., Lee D.-H., Kim D.-M. (2018). A synthetic microbial biosensor for high-throughput screening of lactam biocatalysts. Nat. Commun..

[14] Fu Y., Yeom S.-J., Kwon K.K., Hwang J., Kim H., Woo E.-J., Lee D.-H., Lee S.-G. (2018). Structural and functional analyses of the cellulase transcription regulator CelR. FEBS Lett..

[15] Kwon K.K., Yeom S.-J., Lee D.-H., Jeong K.J., Lee S.-G. (2018). Development of a novel cellulase biosensor that detects crystalline cellulose hydrolysis using a transcriptional regulator. Biochem. Biophys. Res. Commun..

[16] Yang X.A., Zweifach A. (2019). Temperature-Dependent Expression of a CFP-YFP FRET Diacylglycerol Sensor Enables Multiple-Read Screening for Compounds That Affect C1 Domains. SLAS Discov. Adv. Life Sci. RD.

[17] Dai B., Wang L., Wang Y., Yu G., Huang X. (2018). Single-Cell Nanometric Coating Towards Whole-Cell-Based Biodevices and Biosensors. Chem. Sel..

[18] Ganesh I., Vidhya S., Eom G.T., Hong S.H. (2017). Construction of Methanol-Sensing Escherichia coli by the Introduction of a Paracoccus denitrificans MxaY-Based Chimeric Two-Component System. J. Microbiol. Biotechnol..

[19] Lee J.-Y., Sung B.H., Oh S.-H., Kwon K.K., Lee H., Kim H., Lee D.-H., Yeom S.-J., Lee S.-G. (2019). C1 Compound Biosensors: Design, Functional Study, and Applications. Int. J. Mol. Sci..

[20] Ochsner A.M., Müller J.E., Mora C.A., Vorholt J.A. (2014). In vitro activation of NAD-dependent alcohol dehydrogenases by Nudix hydrolases is more widespread than assumed. FEBS Lett..

[21] Kim H.G., Han G.H., Kim D., Choi J.S., Kim S.W. (2012). Comparative analysis of two types of methanol dehydrogenase from Methylophaga aminisulfidivorans MPT grown on methanol. J. Basic Microbiol..

[22] Hernandez K., Bujons J., Joglar J., Charnock S.J., De María P.D., Fessner W.-D., Clapés P. (2017). Combining Aldolases and Transaminases for the Synthesis of 2-Amino-4-hydroxybutanoic Acid. ACS Catal..

