# Supplementary material for: Biosensor-Based Directed Evolution of Methanol Dehydrogenase from Lysinibacillus xylanilyticus
Source: Int J Mol Sci. 2021 Feb 2;22(3):1471. doi: 10.3390/ijms22031471 (PMC7867188; doi:10.3390/ijms22031471)
Supplement: Supplementary file 1 [file ijms-22-01471-s001.pptx]

## Slide 1
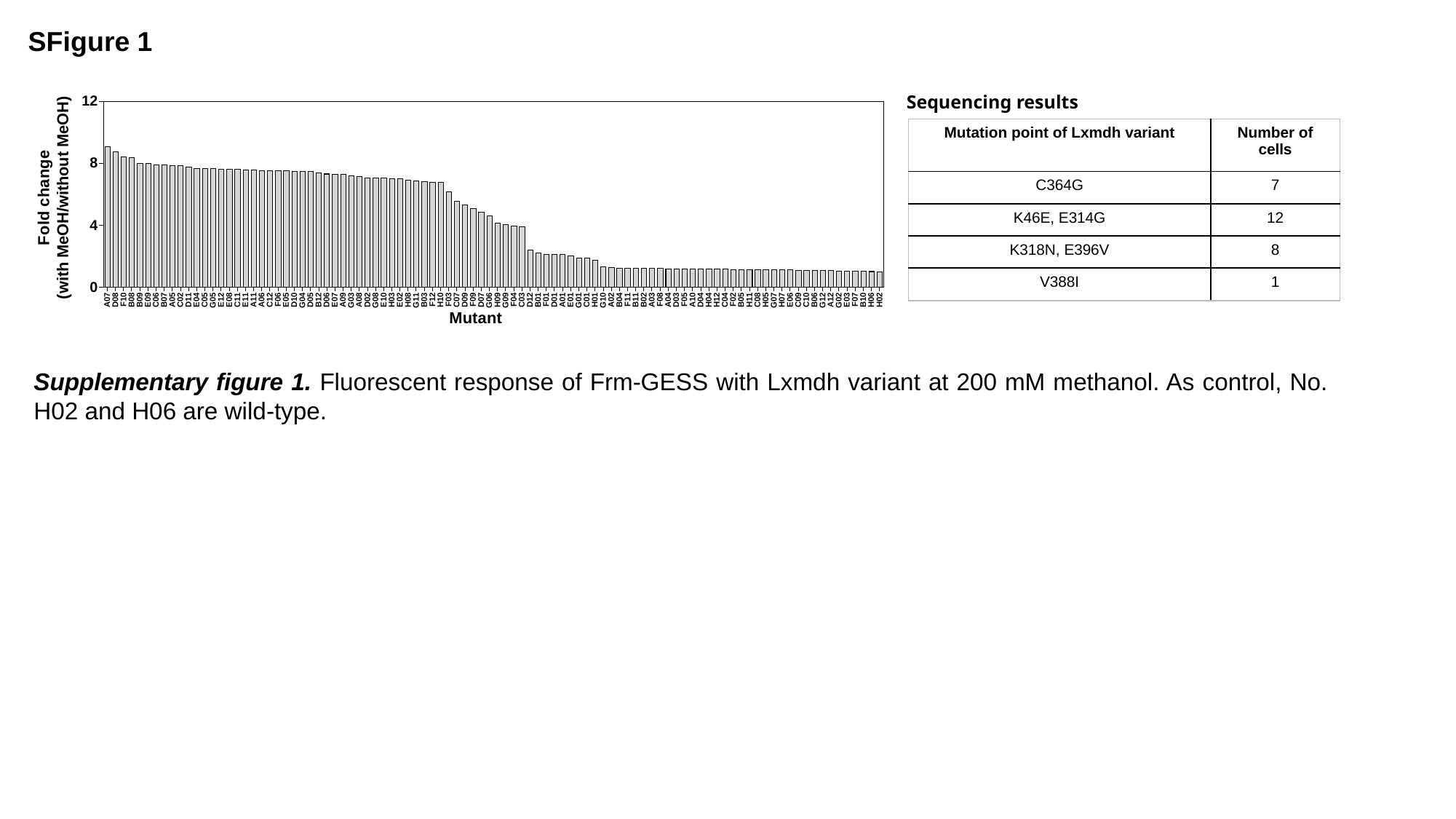

SFigure 1
Sequencing results
| Mutation point of Lxmdh variant | Number of cells |
| --- | --- |
| C364G | 7 |
| K46E, E314G | 12 |
| K318N, E396V | 8 |
| V388I | 1 |
Supplementary figure 1. Fluorescent response of Frm-GESS with Lxmdh variant at 200 mM methanol. As control, No. H02 and H06 are wild-type.

## Slide 2
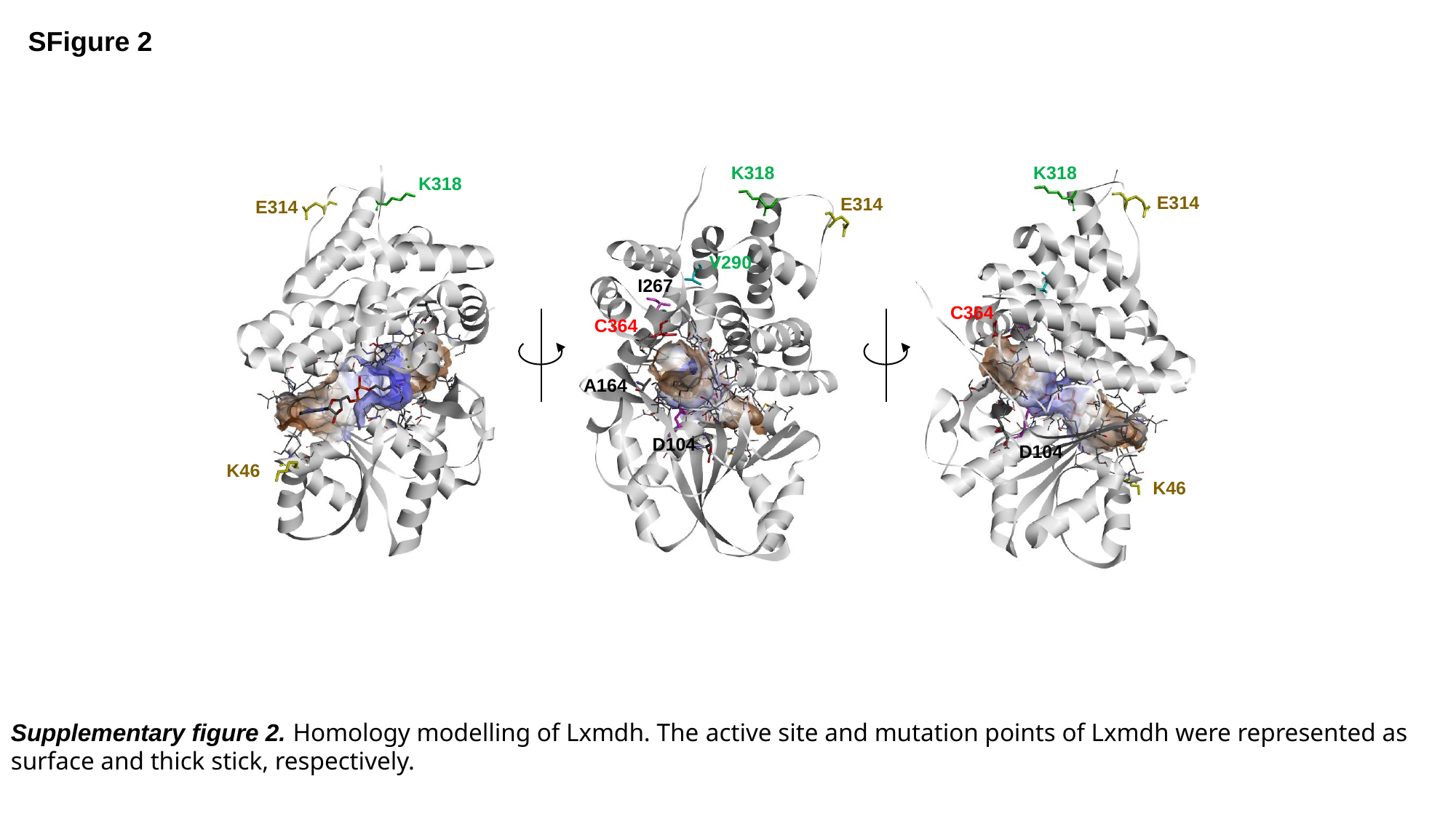

SFigure 2
K318
K318
K318
E314
E314
E314
V290
I267
C364
C364
A164
D104
D104
K46
K46
Supplementary figure 2. Homology modelling of Lxmdh. The active site and mutation points of Lxmdh were represented as surface and thick stick, respectively.

## Slide 3
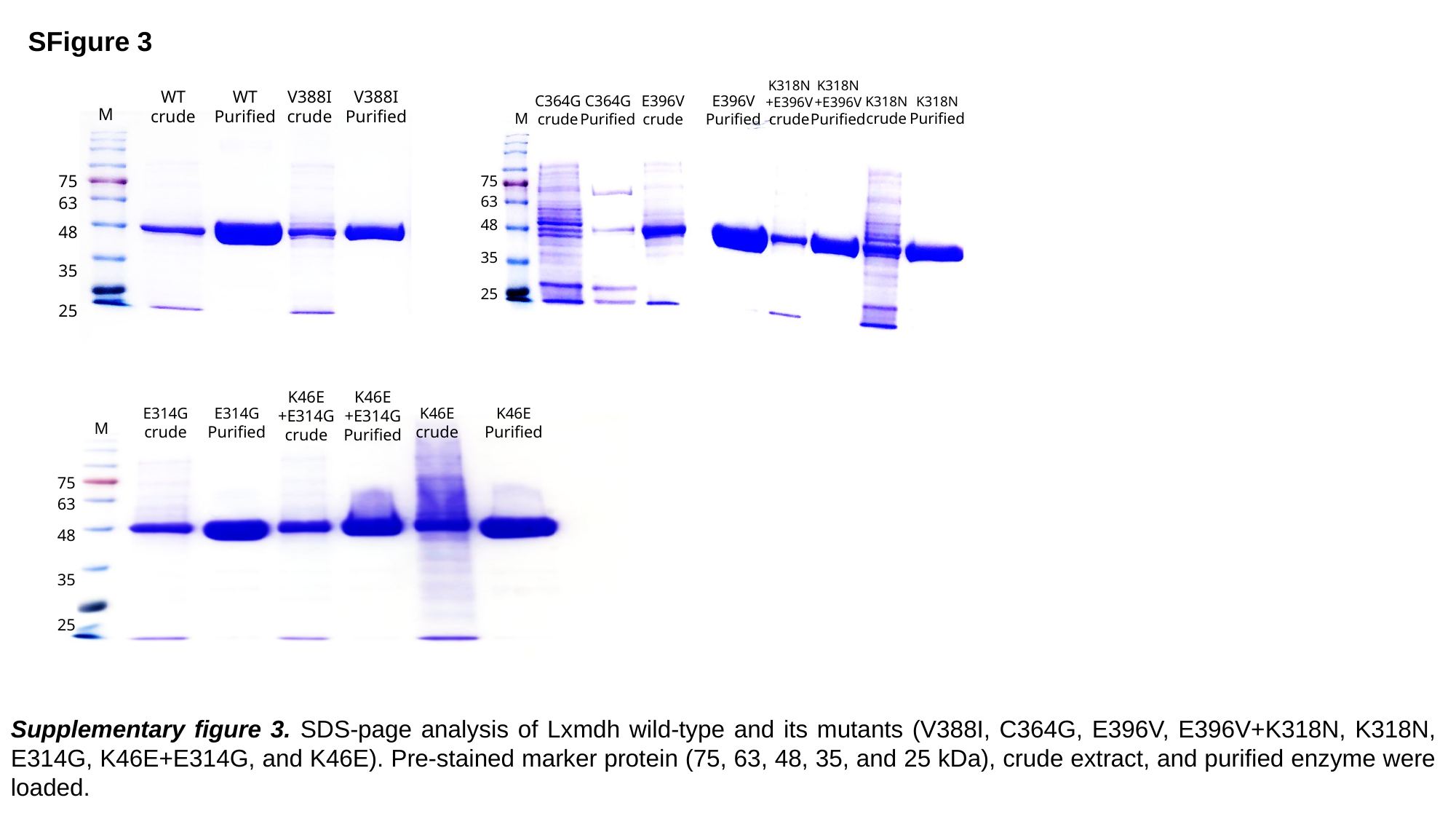

SFigure 3
K318N
+E396V
crude
K318N
+E396V
Purified
E396V
crude
E396V
Purified
C364G
Purified
C364G
crude
K318N
crude
K318N
Purified
M
75
63
48
35
25
WT
crude
V388I
crude
V388I
Purified
WT
Purified
M
75
63
48
35
25
K46E
+E314G
Purified
K46E
+E314G
crude
E314G
crude
E314G
Purified
K46E
crude
K46E
Purified
M
75
63
48
35
25
Supplementary figure 3. SDS-page analysis of Lxmdh wild-type and its mutants (V388I, C364G, E396V, E396V+K318N, K318N, E314G, K46E+E314G, and K46E). Pre-stained marker protein (75, 63, 48, 35, and 25 kDa), crude extract, and purified enzyme were loaded.
